# N2GNet tracks gait performance from subthalamic neural signals in Parkinson’s disease

**DOI:** 10.21203/rs.3.rs-5112726/v1

**Published:** 2024-10-31

**Authors:** Jin Woo Choi, Chuyi Cui, Kevin Wilkins, Helen Bronte-Stewart

**Affiliations:** Stanford University School of Medicine; Stanford University School of Medicine; Stanford University School of Medicine; Stanford University School of Medicine

## Abstract

Adaptive deep brain stimulation (DBS) provides individualized therapy for people with Parkinson’s disease (PWP) by adjusting the stimulation in real-time using neural signals that reflect their motor state. Current algorithms, however, utilize condensed and manually selected neural features which may result in a less robust and biased therapy. In this study, we propose Neural-to-Gait Neural network (N2GNet), a novel deep learning-based regression model capable of tracking real-time gait performance from subthalamic nucleus local field potentials (STN LFPs). The LFP data were acquired when eighteen PWP performed stepping in place, and the ground reaction forces were measured to track their weight shifts representing gait performance. By exhibiting a stronger correlation with weight shifts compared to the higher-correlation beta power from the two leads and outperforming other evaluated model designs, N2GNet effectively leverages a comprehensive frequency band, not limited to the beta range, to track gait performance solely from STN LFPs.

## Introduction

Gait has been noted as one of the heavily researched areas for movement disorders in Parkinson’s disease (PD), as its related symptoms including impairment, slowness of stepping, or freezing may cause devastating incidents such as falling [1]-[3]. One of the possible ways to alleviate these symptoms is through deep brain stimulation (DBS), an implantable system that delivers electrical stimulation to specific brain regions such as subthalamic nucleus (STN) [4]-[6]. By providing a consistent level of stimulation determined by the clinicians to people with PD (PWP) throughout their daily activities, DBS has been adopted in various situations to reduce medication usage and treat motor symptoms [7].

The advancement of DBS allowing sensing capabilities from its leads has paved the way for the therapy to be a closed-loop system, where DBS can adjust its parameters automatically based on sensed neural activity [8], [9]. This recently developed approach is known as adaptive DBS, which adjusts the amount of stimulation in real-time with respect to the motor performance and reduces the exposure of unnecessary amounts of stimulation [10], [11]. One of the common neural signals that adaptive DBS utilize is the local field potential (LFP) recorded from the STNs that reflect the motor state of PWP. Specifically, neural oscillatory activity within the beta frequency band (13–36 Hz) has been known to be associated with movement, including changes in bradykinesia and freezing of gait (FOG) [12]-[16]. Growing evidence has highlighted that adaptive DBS using these biomarkers was effective in alleviating motor symptoms while delivering less stimulation compared to conventional open-loop DBS [17], [18].

Various adaptive DBS algorithms have been proposed to speculate patient’s movement performance from beta-related biomarkers and modulate stimulation accordingly. For instance, beta power had been used in single and dual threshold algorithms, where the stimulation was adjusted depending on one or two clinically defined beta power thresholds, respectively [19]-[21]. Beta burst durations have also been investigated for their feasibility to drive the stimulation, with its consideration that prolonged beta bursts are more associated with motor disability and gait impairment than short bursts [22], [23]. Despite these advancements, using beta power or burst durations with threshold-based algorithms possess some limitations. Due to its nature of neural oscillatory patterns being different between individuals and even within individuals over time, the algorithms using these condensed neural signal features may result in less robust therapy [24]-[26]. Furthermore, these algorithms rely on handcrafted parameters determined through data visualization for selecting which specific neural features to consider. Limiting the utilization of features to such aspects and manually selecting parameters based on summarized information may fail to capture detailed neural characteristics unique to individuals, which may contain important information on real-time gait. These approaches are thus prone to becoming less robust, more subjective, and biased therapies due to their low complexity [27].

In this paper, we introduce Neural-to-Gait Neural network (N2GNet), a deep learning-based data-driven approach capable of predicting gait performance in real-time relying on LFP signals from left and right STNs. Considering that unsupported gait may raise safety concerns such as falls, the LFP data was acquired when participants performed stepping in place (SIP) while being harnessed, and the ground reaction forces from the left and right legs were measured through the corresponding force plates to alternatively assess gait performance [28]. A deep learning model was trained to automatically learn features that correlate the LFP signals with the amount of weight shifts performed by the participants during the SIP task.

## Methods

### A. Participants

The data from eighteen participants who were diagnosed with PD, implanted with bilateral STN DBS leads connected to an implanted investigative neurostimulator (Activa PC + S, Medtronic, PLC), and met the established criteria for dataset formation were used in our study. Participants were instructed to perform the experiment in the off-medication state. This involved stopping long-acting dopamine agonists at least 48 hours, dopamine agonists and controlled release carbidopa/levodopa at least 24 hours, and short acting medication at least 12 hours before testing. All participants gave written consent prior to the study. The study was approved by the Food and Drug Administration with an Investigational Device Exemption and by the Stanford University Institutional Review Board.

### B. Experimental protocol

**Figure 1:**
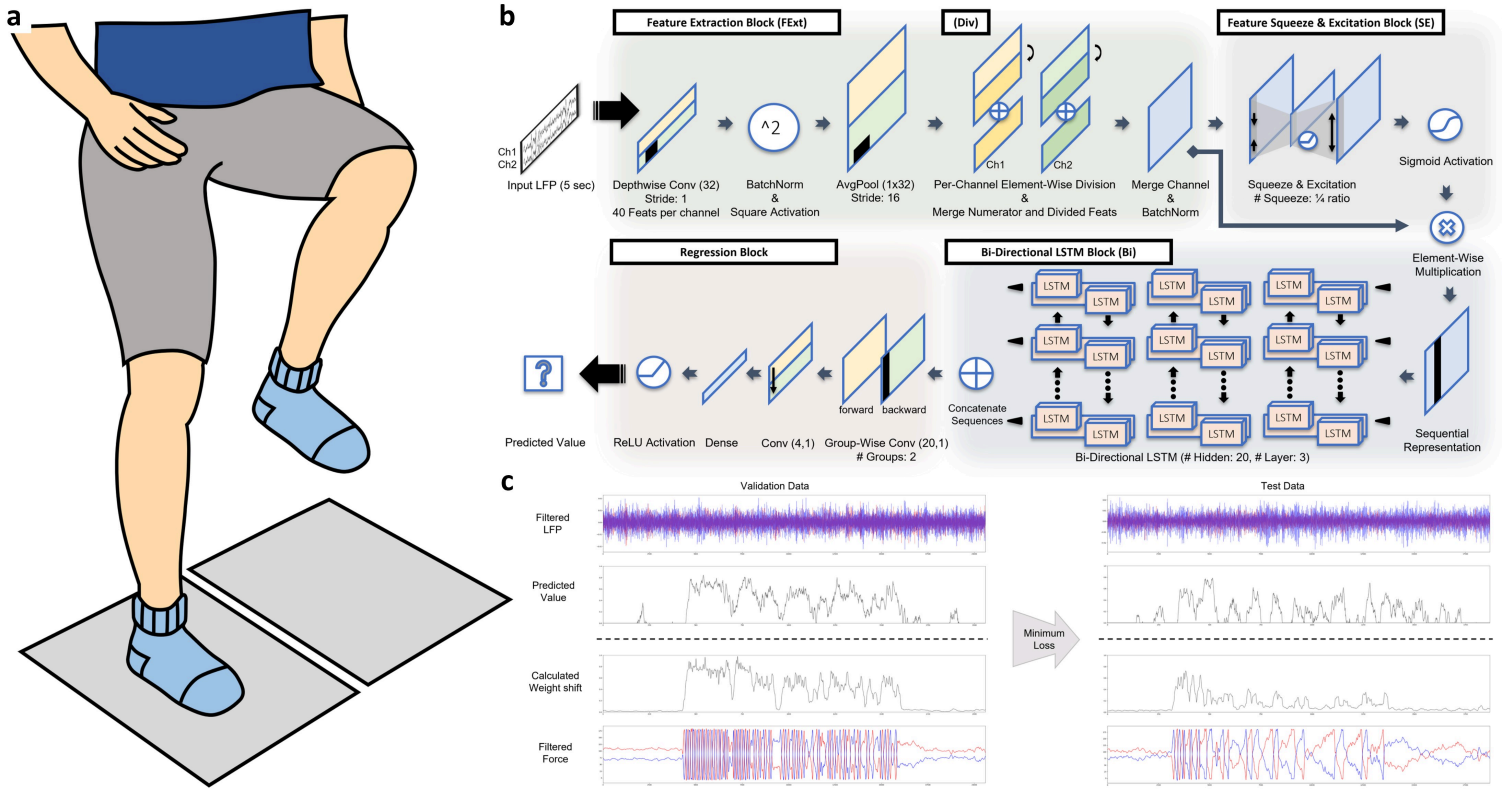
Experiment protocol for N2GNet. a) Data acquisition phase where the LFPs from the two leads were measured while participants were to perform SIP on the two force plates. b) The architecture of N2GNet which takes in 5 second-window LFP signals from the two leads and produces a single outcome representing gait performance. c) Example of N2GNet results when continuously inputting LFP signals retrieved from the two leads. The model trained on the epoch with minimum l1 error rate from the validation dataset was chosen to assess its performance on the test dataset.

Participants performed SIP after DBS had been turned OFF for at least 15 minutes. To prevent any falls, participants wore safety jackets that were securely harnessed to the force plate system. In the beginning of the task, participants were first instructed to stand on two force plates that measure ground reaction force on each foot and were asked to remain as motionless as possible. Participants were then given a start cue and expected to alternatively lift their legs at their own pace for approximately 100 seconds (Fig. 1a). Lastly, participants were given a stop cue and were instructed to stop the movement after. The LFP signals from left and right STNs were recorded simultaneously with the measures from the two force plates.

### C. Data acquisition and dataset formation criteria

Ground reaction forces were measured either using Neurocom (Neurocom Inc., Clackamas, OR, USA) or Bertec (Bertec Corporation, Columbus, OH, USA), which were measured at a sampling rate of 100 Hz and 1000 Hz from the two force plates, respectively. For the ground reaction forces measured at 1000 Hz sampling rate, the data were downsampled into 100 Hz to maintain consistency across data. The LFP signals were initially sampled with a sampling rate of 422 Hz and were downsampled to a 211 Hz sampling rate to train the deep learning model with lighter complexity. A pre-processing step was held on the retrieved force plate data by applying a low-pass filter of 2 Hz, in order to reduce jerky noises that are less likely to be related to gait. The data from the LFP signals were also band-pass filtered with ranges between 8 and 100 Hz to include a comprehensive range of frequency bands that may contain information associated with movement [29], [30].

While most of the participants were involved in multiple visits performing the SIP task, we established criteria for the formation of datasets that were applied to each participant. Given that deep learning models require training, validation, and test datasets for training and evaluating the model, only the data from participants who completed the task at least three times on separate occasions were selected for this study. Specifically, the data acquired from the third most recent visit was used as a training set, the data from the second most recent visit was used as a validation set, and the data from the most recent visit was used as a test set for the model. This usage of visits in chronological order was done to limit the amount of data used for training the model while reflecting the actual usage scenario, where the model would be trained on past data while continuously receiving input of the most recent data for predictions. The datasets from each participant were formed in a way that their recording contacts remained consistent across the three visits. For participants who had their implantable pulse generators re-implanted between their visits, only the data collected before the re-implantation were included to maintain consistency of signal quality across datasets. The data from each visit was also visually inspected prior to the data formation to exclude visits that contained excessive noise not related to the task.

### D. Gait quantification through weight shifts

The force plate data obtained from each task was first rescaled by dividing each data sample by the participant’s weight, which was also retrieved from the two force plates when the participants remained motionless. This adjustment was done prior to quantifying weight shifts such that our measurement would be robust to variations in the participants’ weight over different visits.

As participants distribute their weight over the two force plates when both feet are in contact, the measurements from the force plates are inversely proportional to each other. However, this correlation may not always hold true when one of the feet is off contact during the SIP task. Previous work had demonstrated that there may be cases where changes in force on the contacted force plate may still occur while participants tend to shift back towards the other force plate that is not yet in contact [28]. To take these patterns into consideration, the ground force samples from the plates were merged into a single value per time point by selecting the higher sample of the two, which disregarded samples with lower force measures including those of when the feet was off contact. Subsequently, samples from one of the force plates were mirrored symmetrically with respect to the value that signified half the patient’s weight. Thus, samples having higher values would indicate more weight being placed on one force plate, while samples having lower value would represent more weight being distributed toward the other force plate. The change in force resulting from this merged outcome was quantified over a two-second time window to represent the amount of weight shift during that specific time interval (more details can be seen in Supplementary Fig. 1).

Considering that the task performance of each individual participant varies between visits and the performance also varies among participants, the data was normalized within each participant by mapping the maximum weight shift value of the two-second time window out of the three visits into 1, with a completely motionless state set as 0.

### E. Deep learning model architecture

The architecture of our model is composed of four main blocks in a sequential manner: a feature extraction block, a feature squeeze and excitation block, a bi-directional long short-term memory (LSTM) block, and a regression block as shown in Fig. 1b. The model processes the bandpass filtered LFP signals to generate a single value outcome representing the predicted weight shift that the participant would have exhibited.

#### Feature extraction block

It has been shown that convolutional neural networks (CNNs) are capable of extracting oscillatory features [31], [32]. With inspiration from these previous approaches, our feature extraction block utilized 1-dimensional CNN with batch normalization, square activation function and average pooling to imitate the power measure computation from the inputted two band-pass filtered LFP signals. A 1-dimensional CNN was used in a depthwise manner, where each channel includes features from LFP of a single lead, such that the LFPs from the bilateral STNs are learned separately. Given the presence of aperiodic changes across datasets spanning extended time intervals, we implemented an element-wise division approach that utilizes half of the same-group features from the CNNs to divide the others. This division process was evolved with inspiration from previous studies that considered oscillatory features within specific frequency bands of interest in relation to more general or stabilized signal features [33]–[35]. Both the original numerators and resulting divided outcomes, denoted as original and relative features, were concatenated in a way that both types of features are considered for further model training.

#### Feature squeeze and excitation block

The feature squeeze and excitation block in our model is designed to rescale spectral features, emphasizing those with greater importance. As it is a widely known concept that rescaling features using an encoding and decoding approach such as squeeze and excitation networks or attention modules effectively highlight informative features [36], [37], the feature squeeze and excitation block of our model employs the similar concept. The outcome from the feature extraction block is processed in a way that the feature dimension is encoded and decoded back to its original shape for each length-dimension sample, with ReLU activation function used in between. Specifically, the oscillatory features are squeezed and excited for each temporal slice, with encoding and decoding parameters shared across temporal samples, resulting in an output of the same size as the shape before encoding. The resulting feature after further going through sigmoid activation was multiplied element-wise to the original output of the feature extraction block to rescale the features.

#### Bi-directional LSTM block

A three-layered stacked bi-directional LSTM was employed in our model to consider temporal changes in the LFP features within the provided time window. Recent findings suggest that beta burst duration, in extension to elevated beta power, also closely correlates with motor impairment [18], [23], [33]. Our bi-directional LSTM block was thus used to consider temporal oscillatory changes over time.

#### Regression block

The outcome after the bi-directional LSTM block was subsequently merged to finalize the regression model. By using a groupwise convolutional layer, features from the forward and backward LSTMs were first separately extracted. These features were then merged by sequentially going through convolutional and dense layers, and through ReLU activation function to complete our regression model that inputs 5-second LFP signals to output a single value weight shift prediction.

Note that our N2GNet did not utilize bias for the layers used in the model except for the layers in the feature squeeze and excitation block.

### F. Model training

The overall algorithm was implemented with Python and the deep learning model was designed using Pytorch. The NVIDIA GeForce RTX 4090 GPU was used to train the model, and the adaptive moment algorithm (ADAM) optimization was used with a learning rate of 1e-5. We used the l1 loss function with a batch size of 16. The model was trained with a maximum epoch iteration set to 2000, and the early stopping was held whenever the model did not improve their validation loss for 100 epochs. The trained model from the epoch with the least l1 error rates computed using the validation dataset was selected to evaluate with the testing dataset (Fig. 1c).

The model for our experiment takes the most recent 5-second LFP signals of a particular time point as an input to predict the weight shift over the last 2 seconds. The datasets for training, validation, and testing are formed in a way that they include the last 5-second LFP signals and a single label representing the last 2-second of weight shift at every 0.1 second stride within each task duration. The model was also trained and evaluated in a subject-dependent manner, where the model was trained, validated and tested separately on each participant.

### G. Evaluation and analysis

The length of data varied across tasks and the weight shifts performed by participants, which were rescaled to within the range of 0 and 1 and used as dataset labels, were not uniformly distributed within each task. Taking into account such factors, both mean absolute error (MAE) and mean squared error (MSE), which are two commonly used evaluation metrics for assessing regression performance in machine learning models [27], were computed on both validation and test datasets. This was done in order to provide more comprehensive insights into the performance of the models evaluated in this study, and also with consideration that both validation and test sets were obtained from visits that occurred considerably later than the visits from which the training data were collected. We also conducted correlation analysis using Kendall tau coefficient [38], a non-parametric statistic to measure the association between the two variables, to quantify correlations between the model’s predicted results and the weight shifts. Correlations of each 2-second average beta power computed from the LFPs of the two leads with respect to the weight shifts were also quantified and compared with those between N2GNet’s predictions and the weight shifts to explore the benefits of using N2GNet over beta power.

A model ablation study, a commonly used analysis method for deep learning to investigate how each block composing the final model contributed to its performance [39], [40], was conducted with our N2GNet. A total of seven other models derived from our N2GNet were thus designed, assessed, and compared. The models were named as FExt-Div, FExt-Div + SE, FExt-Div + Bi, FExt-Div + SE + BI, FExt, FExt + SE, and FExt + Bi, depending on which parts of the original N2GNet model were used or neglected (details of the model designs can be seen in Supplementary Fig. 2).

To explore which frequency band signals had more impact on the trained model, we measured the variation ratio of the model’s output concerning different frequency band ranges. Specifically, the following procedure was conducted on each participant’s model to investigate which frequency bands had more influence over other bands:

- The LFP data from the training set was band-pass filtered with each frequency band of interest, and were inputted to the model.
- The model’s outcomes prior to the last ReLU activation from the regression block were hooked for each frequency band of interest, and the variance of these outcomes from the same frequency band of interest were measured.
- Variance measures from different frequency bands were normalized as a variance ratio such that its total resulting from all frequency bands would equal to a value of 1 on each participant.

With the models used in our study producing a single value per input, the variance ratio derived from the resulting values provides insights into which frequency band signals were more influential in producing the final outcome.

The variance ratio in our study were measured using six different frequency bands: delta and theta (∼8 Hz), alpha (8–13 Hz), low-beta (13–20 Hz), high-beta (20–36 Hz), low-gamma (36–70 Hz) and high-gamma (70∼ Hz).

### H. Statistical analysis

For statistical comparisons involving two groups with independent variables, we utilized the Mann-Whitney U test. We also utilized the Wilcoxon signed-rank test for statistical comparisons that involved paired samples. A Kendall tau correlation coefficient analysis was used to quantify the association between two data measures. These statistical tests were performed considering that the number of participants is relatively small (n = 9 for both TD and AR groups, and a total of 18 PD participants), and the non-parametric tests do not assume normal distribution of the data. We reported measures with p-values below a threshold of 0.05 as significant for Mann-Whitney U tests. A threshold of 0.025 (0.05/2) was considered significant for p-values from the Wilcoxon signed-rank tests considering a Bonferroni correction for the two tests conducted, one from validation sets and the other from test sets.

## Results

### A. Participant demographics

A brief overview of the dominant symptom of each participant is shown in Table 1. Participants were divided into two groups depending on their dominant symptoms, a tremor dominant (TD) group and an akinetic rigid (AR) group.

The months the visits took place after the initial programming (IP) of DBS, the duration of task recordings in seconds, and the range of weight shift data labels from each task are also shown for training, validation, and testing datasets in Table 1. The IP visits, which were held for the initial activation of the DBS system, took place a month after the implantation of the DBS leads.

### B. N2GNet performance on neural-to-gait translation

**Figure 2:**
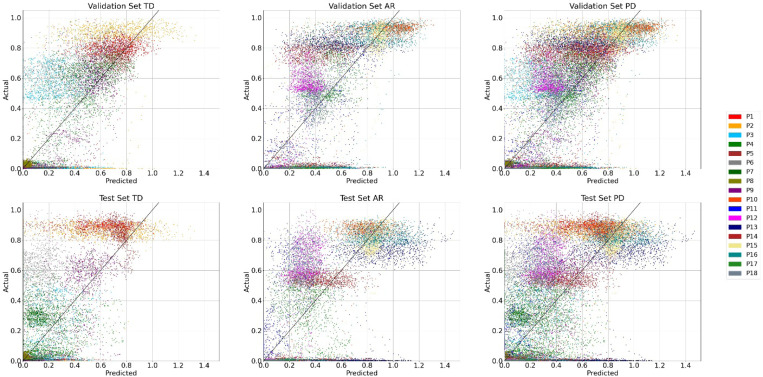
Performance of N2GNet’s prediction for TD group, AR group, and for all PD participants. The x-axis indicates predicted value from our proposed model and the y-axis indicates actual value, which is the weight shift value computed from the force plates.

Figure 2 shows the spread of N2GNet’s predicted results with respect to the actual weight shifts derived from the force plates. The results for TD group participants showed a mean ± standard deviation of 0.132 ± 0.075 for mean absolute error (MAE) and 0.041 ± 0.034 for mean squared error (MSE) with validation datasets, and an average of 0.157 ± 0.083 for MAE and 0.059 ± 0.051 for MSE from test datasets, showing 0.025 and 0.018 increments in the average MAE and MSE, respectively. AR group participants also had an increase in error rates from validation to test datasets, with 0.004 average MAE increments from 0.186 ± 0.088 for validation sets to 0.19 ± 0.093 for test sets, and with 0.009 increments in average MSE from 0.063 ± 0.042 for validation sets to 0.072 ± 0.067 for test sets. No significant differences were observed between the two groups for both MAE and MSE from both validation (MAE with U = 27.0, p = 0.258 and MSE with U = 28.0, p = 0.297, Mann-Whitney U test) and test sets (MAE with U = 31.0, p = 0.436 and MSE with U = 36.0, p = 0.73, Mann-Whitney U test).

The overall N2GNet performance with all PD participants using MAE was 0.174 ± 0.089 for the test datasets, which was 0.015 higher than the average error rate using validation datasets (0.159 ± 0.086). The average MSE was 0.065 ± 0.06 in test datasets, which was also greater by 0.013 than the average MSE from validation datasets, which was 0.052 ± 0.04.

### C. Correlation analysis with N2GNet and with beta power

**Figure 3:**
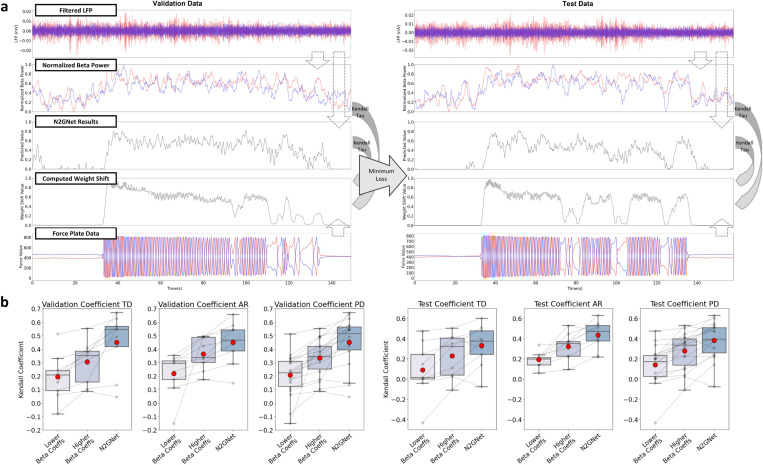
Kendall tau correlation analysis. a) Example Kendall tau correlation comparisons demonstrating the procedures, where the two beta power results acquired separately from the two LFP signals and the prediction results from the N2GNet was referred to the weight shifts computed from the force plates to calculate correlation coefficients. b) Comparisons of correlation results from validation and test sets for TD group, AR group, and for all PD participants. The x axis, from left to right, indicate the lower coefficients out of the two beta power measured from each participant, the higher coefficients of the two, and the coefficients computed with N2GNet results. The gray dots indicate correlation coefficients from each participant, and the dots in red represent the mean value. The boxplots represent first quartile, median, and third quartile for lower, middle, and upper lines in the boxes, respectively, whereas the whiskers represent 1.5 times the IQR extending above the first quartile and below the third quartile.

The correlation results using Kendall tau coefficient between beta power measures and weight shifts, and between predicted values from N2GNet and weight shifts are shown in Fig. 3. As demonstrated in Fig. 3a, average beta power measures over a 2-second interval corresponding to the weight shifts were computed separately for each lead, and the correlation between each beta power measures and the weight shifts was compared to the correlation between N2GNet’s results and weight shifts.

The comparisons between the beta power of lower coefficients, the beta power of higher coefficients, and the coefficients with N2GNet’s predictions show that stronger correlations were exhibited with N2GNet’s predictions than with the beta power from either of the two leads (Fig. 3b). The average Kendall tau coefficient for the TD group from validation sets using N2GNet was 0.45 ± 0.206, which was greater than the average of 0.305 ± 0.147 from the beta power of higher coefficients. N2GNet also exhibited greater Kendall correlation on test sets with 0.33 ± 0.195 compared to the higher-coefficient beta power of 0.229 ± 0.203. As for the AR group, N2GNet’s predictions had higher correlation with the weight shift than the beta power in both validation (0.45 ± 0.148 for N2GNet and 0.364 ± 0.111 for higher-coefficient beta power) and test sets (0.434 ± 0.134 for N2GNet and 0.323 ± 0.123 for higher-coefficient beta power).

Overall with PD participants, N2GNet’s predictions were to have significantly higher correlation with weight shifts compared to the beta power with higher coefficients from the two leads. The prediction results from the model exhibited average correlation coefficient of 0.45 ± 0.179 for validation datasets and 0.382 ± 0.175 for test sets, whereas the beta power with higher correlation exhibited an average coefficient of 0.335 ± 0.134 and 0.276 ± 0.174 for validation and test datasets, respectively (W = 5.0, p = 7.629e-5 for validation sets and W = 20.0, p = 2.808e-3 for test sets, Wilcoxon signed-rank test).

### D. N2GNet model ablation study

**Figure 4:**
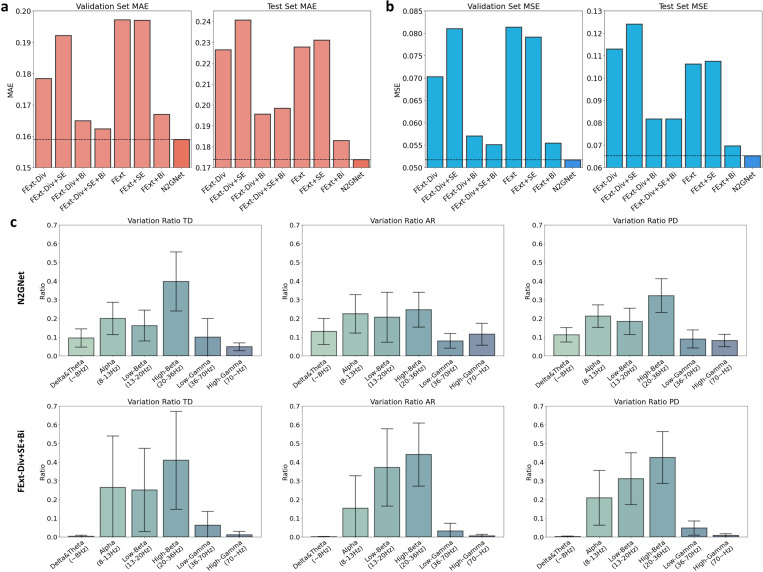
Model ablation study results and analysis. The average error rates using a) MAE and b) MSE on validation and test sets for eight different model designs derived from our proposed model framework. c) Variation ratio results quantifying the impact the data of certain frequency bands of interest affected on the output of the model. Both N2GNet and N2GNet without element-wise division in the feature extraction block (FExt-Div+SE+Bi) were analyzed to explore the effect of considering relative oscillatory features in our model. The error bars represent 95% confidence intervals.

To explore how each block composing our N2GNet affected the prediction performance, a model ablation study was held by eliminating possible block combinations from our N2GNet. The results with our participant data and with seven other possible model designs showed that our N2GNet was able to outperform other models in both MAE and MSE error rates. As can be seen from MAE results in Fig. 4a, N2GNet had less average error rate compared to when bi-lstm block was removed (FExt + SE, validation error: 0.197, test error: 0.231), in the absence of feature squeeze and excitation block (FExt + Bi, validation error: 0.167, test error: 0.183), and in the absence of considering relative features (FExt-Div + SE + Bi, validation error: 0.162, test error: 0.198). Similarly for the average MSE (Fig. 4b), N2GNet had the least error rate on both validation data and test data compared to the absence of bi-lstm block (mean validation error: 0.079, mean test error: 0.108), in the absence of feature squeeze and excitation block (mean validation error: 0.055, mean test error: 0.07), and in the absence of considering relative features (mean validation error: 0.055, mean test error: 0.082). Other possible combinations also exhibited higher error rates than our N2GNet in terms of both MAE and MSE.

Figure 4c further shows variance ratios with respect to different frequency bands for TD group, AR group, and for all PD participants. The analysis was held on our proposed N2GNet and also on the model similar to N2GNet but without the element-wise division process (FExt-Div + SE + Bi) in order to see the effect of considering relative oscillatory features in our model. Results from the model that did not consider relative features exhibited high variance ratios from alpha and beta bands for both TD group (alpha: 0.265 [95% CI: −0.01–0.539], low-beta: 0.25 [95% CI: 0.027–0.473], high-beta: 0.409 [95% CI: 0.147–0.672]) and AR group (alpha: 0.152 [95% CI: −0.021–0.326], low-beta: 0.371 [95% CI: 0.164–0.578], high-beta: 0.44 [95% CI: 0.271–0.609]) compared to other frequency bands of interest. Similarly, N2GNet also exhibited high ratios in these bands for TD group (alpha: 0.199 [95% CI: 0.113–0.286], low-beta: 0.161 [95% CI: 0.079–0.244], high-beta: 0.397 [95% CI: 0.239–0.555]) and AR group (alpha: 0.224 [95% CI: 0.121–0.327], low-beta: 0.206 [95% CI: 0.072–0.339], high-beta: 0.246 [95% CI: 0.153–0.339]). Apart from the alpha and beta bands, N2GNet showed relatively higher ratio on gamma frequency bands (PD group, low-gamma: 0.089 [95% CI: 0.041–0.138], high-gamma: 0.081 [95% CI: 0.049–0.114]) compared to the model without relative features (PD group, low-gamma: 0.047 [95% CI: 0.009–0.085], high-gamma: 0.008 [95% CI: −0.001–0.017]).

We additionally conducted analysis with the models trained and validated with the LFP data band-pass filtered with only the beta band apart from the original signals which were band-pass filtered between 8 to 100 Hz range (Supplementary Fig. 3a and b). When the model was trained and tested with beta-filtered LFP signals, models that included relative features elicited an increased MAE and MSE error rates in validation datasets compared to when original signals were used, resulting in other models to outperform N2GNet with validation datasets for MAE (FExt-Div + Bi: 0.167, FExt-Div + SE + Bi: 0.16, N2GNet: 0.17) and MSE (FExt-Div + Bi: 0.057, FExt-Div + SE + Bi: 0.054, N2GNet: 0.058). Yet, N2GNet still outperformed other models on the test set with its average MAE and MSE of 0.2 and 0.075, respectively. The variation ratio results in Supplementary Fig. 3c further show that the model that excluded relative features was mostly influenced by beta bands, whereas N2GNet still utilized other frequency bands besides the beta range despite the use of beta band-pass filtered signals for its training and evaluation.

## Discussion

The objective of our regression model is to extract neural features that reflect real-time gait of people with PD, solely relying on STN LFPs that do not require additional sensors and can be retrieved directly from the two DBS leads. Our results of mapping neural recordings with the weight shifts measured during SIP tasks exhibited a mean absolute error of 0.174 ± 0.089 and mean squared error of 0.065 ± 0.06 with our proposed N2GNet model using the dataset with continuous labels ranging from 0 to 1. To provide justification for each block composing our model, we further performed a model ablation study employing different deep learning structures that were derived from our proposed architecture. The results demonstrated that our proposed model, in its full structure, was able to achieve the lowest error rates in both MAE and MSE compared to other model designs evaluated in this study. Moreover, the results from Kendall tau correlation analysis elaborate that our model was able to effectively utilize LFPs from the two leads, with its predicted outcomes having greater correlation with the weight shifts from the SIP task compared to either of the beta power measures from the two leads. Most importantly, while our variance ratio analysis provides additional evidence that beta bands contain important information for predicting gait supporting previous studies [12], [15], our additional analysis involving models trained and evaluated with beta-filtered signals along with these results further highlights the importance of taking a wide range of frequency bands into account and not limiting to the beta range. By mapping LFP signals directly into the weight shift values acquired during the SIP task, our N2GNet provides insights into future adaptive deep brain stimulation algorithms that utilize data-driven deep learning methods to predict real-time gait performance, which would be utilized for real-time adjustment of DBS stimulation.

One of the important factors that was considered in designing our N2GNet architecture was the aperiodic component from neural signals. Previous studies had addressed that these aperiodic changes can occur due to age-related cognitive impairments [41] and the severity of motor symptoms [35]. Furthermore, aperiodic changes could be observed in PWP throughout 18 months of their visits after the implantation of DBS [24], which do not necessarily reflect direct movements of PWP. These aperiodic activity changes may not only degrade the performance of low-complexity algorithms that utilize handcrafted thresholds, but may also crucially influence machine learning approaches with designs that are prone to overfitting. The use of relative oscillatory features computed through the element-wise division process, which was held in our feature extraction block, was thus employed to have the model consider not only the oscillations in the signals but also the changes in oscillatory features with respect to general signal trends. This matter is designed with inspiration from previous studies that normalized beta LFP signals with signals from the gamma band, which are more stabilized and less prone to artifacts [33], [34]. Throughout our results, we were able to confirm that the N2GNet benefited from this approach by exhibiting lower error rates compared to when the division procedure was removed.

In line with the improvement of performance by jointly considering relative oscillatory features, our results also highlight the drawbacks of relying mostly on beta signals for designing adaptive DBS algorithms. The results of comparing the variation ratios of different frequency bands of interest showed that N2GNet exhibited a comparatively even distribution across different bands and achieved lower error rates than the model that did not consider relative features. Furthermore, all our models showed increased error rates on the test set when beta-filtered LFPs were used for training and testing the model, compared to when the original signals of 8 to 100 Hz band ranges were used. These results support our claim that relying solely on the beta band may weaken the robustness of the model. It is also worth noting that the models containing the element-wise division process had smaller error rate differences between the validation and test sets compared to the models without division. These factors indicate that including relative features through our division process, along with considerations of other frequency bands beyond the beta band, were more robust over time, as there existed time gaps between the acquisition of validation and test data. With the fact that the only difference between the models in terms of their architectures is the existence and absence of element-wise division within the feature extraction block, the results in our study demonstrates that taking the entire spectrum of signals into account and considering relative oscillations between different frequency bands may enhance the algorithm’s performance, especially when the algorithm needs to maintain its performance over an extended period.

Surprisingly throughout our model ablation study and the variance ratio analysis from Fig. 4c and Supplementary Fig. 3c, we discovered that the N2GNet was able to utilize signals outside the band-pass filtered range, while the other model without element-wise division exhibited its ratio most entirely within the filtered range. We suspect the cause of such results to be from the ability of the band-pass filter not completely eliminating signals outside desired band ranges [42], [43]. Our N2GNet’s utilization of relative features through element-wise division tends to consider the ratio between two different oscillatory features rather than the direct scale, indicating that the model’s relative feature computation would require additional oscillatory feature to act as reference to the original oscillatory feature. This may have led our N2GNet to consider signals outside of filtered band ranges during model training, even though their scales were greatly reduced through filtering.

In addition to the aforementioned phenomenon identified through the variance ratio analysis, it is also important to stress that our variation ratio measure serves as an indication of which frequency band signals caused more variability in the resulting prediction outcomes. Having higher variance in a specific frequency band does not necessarily imply a stronger theoretical correlation with gait, as our variance ratios are completely determined from the perspective of the trained model itself. This should be reminded even stronger for our N2GNet which utilize relative oscillatory features, as the model has the potential to use frequency ranges that are unrelated to gait as a reference feature. Thus, our variation ratio results have limitations and should be interpreted carefully. Rather than as a thorough interpretational method, our variance analysis should serve as an indication that demonstrated improvements in prediction performance by taking into account wide frequency ranges.

Our N2GNet exhibited prediction performance without significant difference between the TD and AR groups in our study, suggesting that the model may be robust for different subtypes of PD. This may be an interesting result as TD typically elicits less changes in beta than AR during movement [44], [45], while both TD and AR can still exhibit gait impairment and FOG [46]. Note that one of the factors that caused the average MAE and MSE from the validation and test datasets to be relatively smaller in the TD group was due to one of the participants exhibiting freezes throughout the entire task from the visits corresponding to the validation and test datasets (Participant 8). The participant did not experience major freezes during the task in the visit that was used as training data, resulting in low MAE and MSE results for that particular participant.

Through our study, we aimed to address the potentials of using deep learning-based methods for predicting real-time gait of PWP solely with STN LFPs, which can further be extended into adaptive DBS algorithms in the near future. Contrary to previous algorithms that utilize compressed features such as beta power or beta burst duration, the ultimate goal of our work is to have features automatically learned within the model, providing optimal and personalized therapy for each individual patient. Unlike current parameter determination procedures which involve individuals performing multiple movement tasks and clinicians repeatedly observing, evaluating movements, and tuning necessary parameters throughout summarized information without taking detailed features into account, our N2GNet is geared towards automatically adjusting the parameters with only a few SIP recording trials. To also resemble practical usage of our proposed model, we evaluated our model in a way that the training, validation, and test datasets were determined in a chronological order, with each dataset containing a single SIP recording session from a visit.

While this is merely a step towards bringing a deep learning-based method for adaptive DBS, limitations exist when it comes to the practical usage of N2GNet in real-life adaptive DBS systems, and future works can be conveyed to enhance our algorithm. Our model is trained to translate LFP signals into weight shifts that resemble gait, however, these weight shifts may not always correlate with the ideal amount of stimulation needed for actual walking. For instance, it is possible that certain PD-related symptoms, such as shuffling with its light but frequent weight shifts, may rather increase the amount of weight shifts and result in a decrease of stimulation when the model is trained with weight shifts as labels. Although we aimed to filter such possibilities by applying a low-pass filter to the force plate data at 2 Hz, further investigation of whether such a pre-processing method would effectively eliminate these instances should be conducted. The nature of LFP signals having aperiodic components serves as another limitation, as our method of extracting relative oscillatory features is still not completely independent from the aperiodic neural activity. Improvements in prediction performance can be held and designing lighter models that reduce computational cost should also be conveyed in order to embed the algorithm into the DBS system. Lastly, our current N2GNet architecture does not consider any possible influences from DBS stimulation. The model should consider both direct contamination of signals from stimulation and indirect physiological consequences that could alter oscillatory features in any way. Further works to enhance the model’s robustness to stimulation can be carried out in advance for the practical application of N2GNet in real-life DBS systems.

## Conclusion

In this study, we developed a novel deep learning-based model that relies only on local field potentials from the subthalamic nucleus to predict real-time gait performance of people with Parkinson’s disease. Our N2GNet achieved the lowest error rate in prediction by using relative oscillatory features, which were obtained by simply adding an element-wise division process that resulted in considering a broader range of signal frequency bands. Compared to other model designs from our model ablation study, N2GNet exhibited greater robustness across both validation and test datasets, which were composed of data acquired considerably after than those from training datasets. Our study not only shows the potential of applying deep learning for adaptive DBS algorithms in keeping track of real-time gait performance with local field potentials but also emphasizes the benefits of taking overall frequency ranges, beyond just utilizing beta bands known to be associated with movement, into account for gait prediction.

## Supplementary Material

Supplement 1 Table 1 is available in the Supplementary Files section.

## Data Availability

The datasets used for the current study are not publicly available, but may be available to qualified researchers from the corresponding author upon reasonable request.
