# Supplementary material for: N2GNet tracks gait performance from subthalamic neural signals in Parkinson’s disease
Source: Res Sq. 2024 Oct 31:rs.3.rs-5112726. Preprint. [Version 1] doi: 10.21203/rs.3.rs-5112726/v1 (PMC11581115; doi:10.21203/rs.3.rs-5112726/v1)
Supplement: Supplement 1 — Table 1 is available in the Supplementary Files section. [file NIHPPRS5112726V1-supplement-1.pdf]

Supplementary Files

This is a list of supplementary files associated with this preprint. Click to download.

- N2GNetSupplementary.docx
- floatimage2.jpeg
